# Polipharmacy and Multimorbity in Oncological Patients: An Open Challenge

**DOI:** 10.3390/curroncol28010030

**Published:** 2021-01-04

**Authors:** Claudia Loreti, Letizia Castelli, Daniele Coraci, Augusto Fusco, Silvia Giovannini, Luca Padua

**Affiliations:** 1 UOC Neuroriabilitazione ad Alta Intensità, Fondazione Policlinico Universitario A. Gemelli IRCCS, 00168 Rome, Italy; letizia.castelli@gmail.com (L.C.); danielecoraci@aol.com (D.C.); augusto.fusco@policlinicogemelli.it (A.F.); luca.padua@unicatt.it (L.P.); 2 Department of Neurosciences, Università Cattolica del Sacro Cuore, 00168 Rome, Italy; 3 UOC Riabilitazione e Medicina FIsica, Fondazione Policlinico Universitario A. Gemelli IRCCS, 00168 Rome, Italy; silvia_giovannini@yahoo.it; 4 Department of Geriatrics and Orthopaedics, Università Cattolica del Sacro Cuore, 00168 Rome, Italy

**Keywords:** drug therapy, multimorbidity, personalized medicine

## Abstract

Kirkham and colleagues presented an original study about cancer survivors in Canadian population and reported that the odds of several cardiovascular disease risk factors are higher among middle-aged. Several risk factors are connected to a toxic lifestyle and are associated with cardiovascular diseases and general health status. The paper is very relevant in managing oncological patients. A particular attention should be given to some anamnestic data about the presence of other pathologies (as self-reported diabetes and hypertension) and drug therapy with particular consideration of angiotensin-converting enzyme inhibitors that present a protective action against cardiovascular events and reduce the incidence of type II diabetes. In order to identify and intervene on risk factors, clinicians should depict the pharmacological therapy taken by the study population, assuming that in the elderly this may be potentially protective on cardiovascular risk profile compared to younger cancer survivors.

Dear Editor,

We have read with high consideration and interest the article, “Age-dependent increased odds of cardiovascular risk factors in cancer survivors: Canadian Longitudinal Study on Aging cohort”, by Kirkham and colleagues and recently published in Your prestigious journal [1].

In the study, the authors reported that the odds of several cardiovascular disease risk factors are higher among middle-aged, but not older, cancer survivors relative to the general Canadian population [1]. Analyzing risk factors, the authors recognize several elements that are imputable to a toxic lifestyle, such as decreased fruits and vegetables intake, smoking status, reduced physical activity and the onset of diabetes [1]. The authors point out that all these variables are strongly associated with risk factors in considered population for cardiovascular disease and general health. In particular, our attention was focused on the association between diabetes and the risk of cardiovascular diseases. Self-reported diabetes was the cardiovascular risk factor with the highest odds, along with hypertension, being able to increase the risk of death from peripheral vascular disease by 20–30%. This paper is particularly relevant for the management of oncological patients. An early diagnosis and a consequent management of diabetes (and its peripheral vascular complications) are particularly important in oncological patients who present high frailty conditions [2]. 

From this perspective, it would be clinically relevant and interesting for medical research to know the drug therapy that the study population is taking. In fact, angiotensin-converting enzyme (ACE) inhibitors have been demonstrated to protect against cardiovascular events and reduce the incidence of type II diabetes [2]. However, the magnitude of the therapeutic effects of ACE-inhibitors outweighs that expected based on their anti-hypertensive action. For example, it has been reported that a six-month treatment with an ACE inhibitors increases systemic levels of total Insulin-like Growth Factor 1 (IGF-1) and Insulin-like Growth Factor Binding Protein 3 (IGFBP-3) in older adults with high cardiovascular risk profile with several potential beneficial effects [3]. 

The authors also reported that individuals currently having cancer were less likely to be active than were age-matched adults [1]. For this reason, ACE inhibitors positive effects on skeletal muscle have been clearly documented even in late-life [4]. It has also been documented the beneficial effect of ACE inhibitors administration on body composition and on perceived quality of life in elderly population [5].

Therefore, in addition to knowing the clinical history and lifestyle habits of cancer survivors, as the authors point out [1], in order to identify and intervene on risk factors, it would therefore be important to know the pharmacological therapy taken by the study population assuming that in the elderly this may be potentially protective on cardiovascular risk profile compared to younger cancer survivors. 
